# Chronotype of Lung Fluid Levels in Patients with Chronic Heart Failure

**DOI:** 10.3390/jcm11102714

**Published:** 2022-05-11

**Authors:** Yohei Ueno, Teruhiko Imamura, Nikhil Narang, Koichiro Kinugawa

**Affiliations:** 1 Second Department of Internal Medicine, University of Toyama, Toyama 930-0194, Japan; fef6ge@gmail.com (Y.U.); kinugawa-tky@umin.ac.jp (K.K.); 2 Advocate Christ Medical Center, Oak Lawn, IL 60453, USA; nikhil.narang@gmail.com

**Keywords:** congestion, hemodynamics, ReDS, circadian rhythm

## Abstract

Background: The variation in lung fluid levels dependent on chronotype in patients with chronic heart failure is unclear. Remote dielectric sensing (ReDS^TM^) is a novel non-invasive system to quantify the lung fluids, which may correlate to intracardiac filling pressures. We aimed to understand the variation in ReDS measurements by chronotype in patients with chronic heart failure. Methods: The patients who were hospitalized for heart failure exacerbations between November 2021 and March 2022 were prospectively included. ReDS values were measured at clinically stable conditions at the following three time points during the day: 5:00 (morning), 12:00 (noon), and 21:00 (night) (manufacture-recommended reference of ReDS value: between 25% and 35%). Results: Twelve patients were included. The median age was 84 (75, 90) years and four patients (33%) were men. The median plasma B-type natriuretic peptide was 235 (178, 450) pg/mL. The median ReDS value was 38% (23%, 41%) in the morning. The ReDS value decreased significantly at the noon measurement, down to 28% (23%, 29%) (*p* = 0.005) and again increased significantly at the night measurement, up to 31% (27%, 42%) (*p* = 0.002). The patients were clinically stabilized during the observational period. Conclusions: the lung fluid level varied considerably in patients with chronic heart failure following clinical stabilization.

## 1. Introduction

Variations in human physiology, as exhibited in changes in blood pressure, heart rate, and metabolism, occur over the course of sleep-wake cycles with specific regulation by circadian sleep-wake cycles [1]. Patients with heart failure have unique chronotypes, which may also contribute to changes in day–night vital signs to intracardiac filling pressures, although rigorous data evaluating this question is lacking [2]. For example, the timing of onset of cardiovascular disease, including heart failure, stroke, and acute coronary syndrome, seems to have specific unique trends during the day.

Pulmonary congestion may worsen later in the day, as the effect of neurohormonal agents wane for those who take medical therapy in the morning, whereas pulmonary congestion may be at a nadir mid-day, following the true effect of medical therapy and drops in morning cortisol levels. Of note, changes in lung fluid levels, which often correlate with intracardiac filling pressures and degree of pulmonary congestion [3], by chronotype remains uncertain in patients with chronic heart failure. Thus, we hypothesized that there may be a unique chronotype that explains these potential differences between day and night. Considerable day–night variation data in these important clinical parameters may better inform the clinician on appropriate therapeutic interventions.

**Figure 1:**
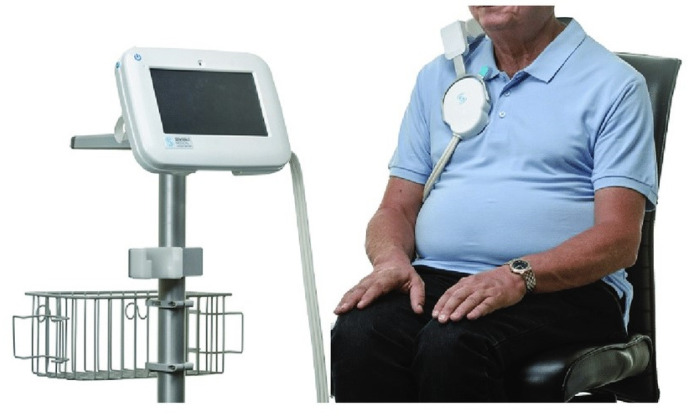
A ReDS system, which consists of a monitor and a sensor unit.

The remote dielectric sensing (ReDS^TM^, Sensible Medical Innovations Ltd., Netanya, Israel) system is a novel electromagnetic-based method to quantify lung fluid levels with robust inter-rater and intra-rater reliability (Figure 1) [4,5,6]. According to the previous studies, ReDS values had an acceptable correlation with pulmonary capillary wedge pressure, measured by right heart catheterization and lung fluid amounts, calculated by high-resolution computed tomography in patients with chronic heart failure [7,8].

In this prospective study, we measured ReDS values successively during the day in patients with chronic heart failure as a primary concern, to better understand the chronotype of lung fluid levels.

## 2. Methods

### 2.1. Participant Selection

The patients who were hospitalized to treat decompensated heart failure were considered for inclusion in this prospective study, following clinical stabilization by the inpatient treatment team. Following comprehensive informed consents, ReDS values were measured at the following three times during the day: 5:00 (morning), 12:00 (noon), and 21:00 (night). In the morning, the ReDS value was measured under fasting conditions before taking any medications, including diuretics.

### 2.2. ReDS System

ReDS values were measured at sitting position, according to the manufacture-recommended methodology [4]. ReDS employs low-power electromagnetic signals emitted between two sensors embedded on the wearable devices (Figure 1). The manufacture-recommended reference range is between 25% and 35%.

### 2.3. Statistical Procedures

All continuous data are presented as median with interquartile range. Categorical data are presented as numbers and percentages. The trends of the ReDS values were analyzed using the Friedman test and post-hoc Wilcoxon signed-rank test. Statistics were performed using SPSS Statistics 23.0 software (IBM Corp, Armonk, NY, USA). Two-sided *p* values less than 0.05 were considered significant.

## 3. Results

### 3.1. Baseline Characteristics

A total of 12 patients who were hospitalized for decompensated heart failure were included (Table 1). The median age was 84 (75, 90) years and four patients (33%) were men. Half of the patients had a history of previous heart failure hospitalization; no patient had a history of chronic obstructive pulmonary disease. The median plasma B-type natriuretic peptide was 235 (178, 450) pg/mL and left ventricular ejection fraction was 59% (56%, 62%). Half of the included patients were on diuretics prior to admission.

### 3.2. Chronotype of ReDS Values

**Figure 2:**
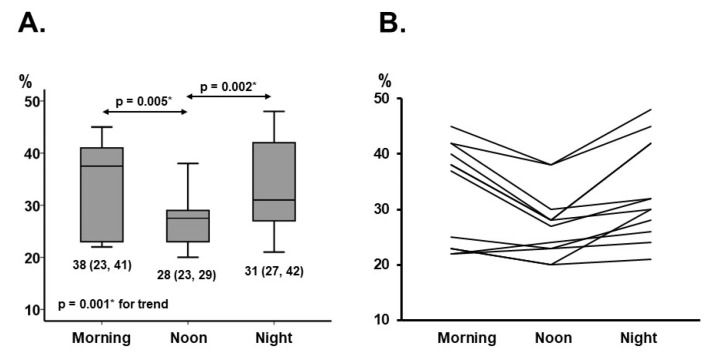
Trends of ReDS values during the day expressed as median values (**A**) and individual absolute values (**B**). Trends were assessed using Friedman test and post-hoc Wilcoxon signed-rank test. * *p* < 0.05.

The trend of the ReDS values varied considerably during the day, following initial clinical stabilization (*p* = 0.001; Figure 2A,B). In the early morning, the median ReDS value was 38% (23%, 41%). The ReDS value decreased significantly to 28% (23%, 29%) at noon (*p* = 0.005), and then increased significantly at the night measurement up to 31% (27%, 42%) (*p* = 0.002). The systolic blood pressure and heart rate showed a similar trend. The systolic blood pressure was 129 (115, 38) mmHg, 121 (117, 130) mmHg, and 125 (118, 128) mmHg (*p* = 0.035). The heart rate was 79 (69, 88) bpm, 76 (68, 77) bpm, and 77 (73, 82) bpm (*p* = 0.022).

Seven patients had a ReDS value of >35% in the early morning, which had no significant association with the baseline characteristics (*p* > 0.05 for all).

## 4. Discussion

In this preliminary proof-of-concept prospective study, we observed a unique chronotype of lung fluid levels, as quantified by the ReDS system. Overall, lung fluid levels were highest in the morning, with subsequent decreases during the day and an uptrend at night.

### 4.1. Chronotype of Cardiovascular Parameters

In patients with chronic heart failure, sympathetic tone can be abnormally elevated at night, as observed with incremental changes in blood pressure and heart rate [9]. Additionally, endothelial dysfunction in patients with chronic heart failure may further increase peripheral vascular constriction. The activation of plasma arginine vasopressin and renin-angiotensin systems may be exaggerated during the night hours, which may increase stressed blood volume [10]. These mechanisms may explain the potential increase in pulmonary congestion, which in turn triggers heart failure exacerbation [2]. Our findings support this theory, as we observed higher ReDS values in both the night and early morning measurements. Consistently, using another modality impedance cardiography, nocturnal whole thoracic volume overload, instead of pulmonary congestion, was recently observed [11].

### 4.2. Other Factors Associated with Chronotype of Lung Fluid Levels

Another trigger of acute heart failure during the night measurement is body position. The supine position increases venous return and worsens pulmonary congestion in patients with chronic heart failure, as we also demonstrated with the ReDS system measurements [12]. Of note, ReDS values were measured in this study at sitting position, instead of spine position. The patients also received their medications, including diuretics, following the ReDS measurements in the morning. This would be another reason for lower ReDS values during the day time.

### 4.3. Study Limitations

This is a preliminary proof-of-concept study and has several limitations. This study consists of a small sample size. We included patients with chronic heart failure following clinical stabilization and most of the ReDS values were within the manufacture-suggested normal range. The findings in this study are not applicable to patients with acute heart failure requiring ongoing medical optimization. Furthermore, the ReDS values were measured in a sitting position while patients were awake, and not recorded during periods of sleep.

### 4.4. Conclusions

The ReDS system is a novel and promising device to quantify the amount of lung fluid with a reference between 25% and 35%. We observed clinically significant variations in the values between 28% (noon) and 38% (morning) during the observed measurement period. These unique time-varying differences may inform the clinician as to when therapies may be dosed to ensure longer periods of clinical stability.

## Figures and Tables

**Table 1:** Baseline characteristics.

	N = 12
Demographics	
Age, years	84 (75, 90)
Men	4 (33%)
Body mass index	21.7 (20.2, 22.9)
Comorbidity	
Hypertension	10 (83%)
Diabetes mellitus	5 (42%)
Atrial fibrillation	3 (25%)
Chronic obstructive pulmonary disease	0 (0%)
Ischemic heart disease	6 (50%)
History of stroke	2 (17%)
Peripheral artery disease	0 (0%)
History of previous heart failure admission	6 (50%)
Laboratory data	
Hemoglobin, g/dL	11.7 (10.4, 13.2)
Serum albumin, g/dL	3.6 (3.2, 3.9)
Serum sodium, mEq/L	139 (137, 141)
Serum potassium, mEq/L	4.3 (3.8, 4.6)
Serum total bilirubin, mg/dL	0.5 (0.5, 0.7)
Estimated glomerular filtration ratio, mL/min/1.73 m^2^	47 (39, 62)
Serum C-reactive protein, mg/dL	0.3 (0.1, 0.9)
Plasma B-type natriuretic peptide, pg/mL	235 (178, 450)
Echocardiography	
Left ventricular end-diastolic diameter, mm	51 (44, 53)
Left ventricular ejection fraction, %	59 (56, 62)
Mild or greater mitral regurgitation	3 (25%)
Mild or greater tricuspid regurgitation	2 (17%)
E/A ratio	0.74 (0.56, 1.15)
Inferior vena cava diameter expiratory/inspiratory, mm	11 (8, 15)/6 (4, 7)
E/e’ ratio	12.4 (10.1, 14.3)
Medication	
Beta-blocker	9 (75%)
Renin-angiotensin system inhibitor	7 (58%)
Mineralocorticoid receptor antagonist	4 (33%)
SGLT2 inhibitor	4 (33%)
Diuretics	6 (50%)

## Data Availability

Data are available upon reasonable reasons from the corresponding author.
